# Minimization of Torque Deviation of Cylinder Deactivation Engine through 48V Mild-Hybrid Starter-Generator Control

**DOI:** 10.3390/s21041432

**Published:** 2021-02-18

**Authors:** Hyunki Shin, Donghyuk Jung, Manbae Han, Seungwoo Hong, Donghee Han

**Affiliations:** 1 Eco-Vehicle Control Design Team, Hyundai KEFICO Corporation, Gunpo 15849, Korea; hyunki.shin@hyundai-kefico.com; 2 Department of Automotive Engineering, Hanyang University, Seoul 04763, Korea; jdh1776@hanyang.ac.kr; 3 Department of Mechanical and Automotive Engineering, Keimyung University, Daegu 42601, Korea; 4 Research & Development Division, Hyundai Motor Company, Hwaseong 18280, Korea; swhong@hyundai.com (S.H.); dh@hyundai.com (D.H.)

**Keywords:** combustion model, cylinder deactivation, engine modeling, in-cylinder pressure, SI turbulent flame model, 48V mild-hybrid starter-generator

## Abstract

Cylinder deactivation (CDA) is an effective technique to improve fuel economy in spark ignition (SI) engines. This technique enhances volumetric efficiency and reduces throttling loss. However, practical implementation is restricted due to torque fluctuations between individual cylinders that cause noise, vibration, and harshness (NVH) issues. To ease torque deviation of the CDA, we propose an in-cylinder pressure based 48V mild-hybrid starter-generator (MHSG) control strategy. The target engine realizes CDA with a specialized engine configuration of separated intake manifolds to independently control the airflow into the cylinders. To handle the complexity of the combined CDA and mild-hybrid system, GT-POWER simulation environment was integrated with a SI turbulent combustion model and 48V MHSG model with actual part specifications. The combustion model is essential for in-cylinder pressure-based control; thus, it is calibrated with actual engine experimental data. The modeling results demonstrate the precise accuracy of the engine cylinder pressures and of quantities such as MAF, MAP, BMEP, and IMEP. The proposed control algorithm also showed remarkable control performance, achieved by instantaneous torque calculation and dynamic compensation, with a 99% maximum reduction rate of engine torque deviation under target CDA operations.

## 1. Introduction

Regulations for energy and exhaust emissions are major challenges in the research and development of powertrains. To overcome such challenges, cylinder deactivation (CDA) technology has drawn attention as a way to make engine displacement flexible; the process involves deactivating a several cylinders under low-load conditions [1,2,3]. The CDA deactivates several cylinders with wider throttle openings for compensating the required torque. Wider throttle opening reduces pumping loss and thus improves fuel economy [4,5,6,7,8,9,10,11].

Hybridization also has been demonstrated as a promising method to improve the fuel efficiency and reduce exhaust emission from the internal combustion engine [12,13,14]. An optimization of several parameters via a genetic algorithm minimizes the brake specific fuel consumption and nitric oxide emission [15]. The CDA combined with a hybridization improves fuel efficiency by activating only three cylinders out of the six-cylinder engine [16].

Aside from the consideration of the fuel efficiency and exhaust emissions, the successful implementation of CDA should suppress adverse effects such as noise, vibration, and harshness (NVH). Such NVH problem results from torque deviation between cylinders when activating CDA because activated cylinders must provide more torque to maintain vehicle driving performance. The absence of combustion in the deactivation sequences reduces, therefore, the number of power strokes per revolution and extends the interval between the maximum torque pulses, making it difficult to deliver consistent and smooth brake torque from the engine [17,18].

Overcoming the NVH problems of CDA has been challenging in terms of cost of additional components, calibration efforts, and limited control accuracy. Such drawbacks hinder the realization of CDA in the commercial market. The conventional approach for mitigating NVH problems of CDA engines was to use advanced hardware systems, such as additional torsional dampers and suitable engine mounts [19,20]. Although this method alleviates vibration of the drivetrain, cost and weight increased due to the additional hardware equipment. Unlike such hardware systems used to cope with NVH problems, the Dynamic Skip Fire (DSF) system improves NVH performance by selecting diverse firing sequences through independent control of individual cylinders [21,22,23,24,25]. The high degree of freedom to activate DSF operation increases calibration efforts. To complement this DSF system, an additional motor generator unit was integrated with a 48V electric hybridization [26]. The combined system presented the ability to smooth torque pulsation based on mild-hybrid torque assist; however, the open loop scheme makes it difficult to guarantee accurate control performance without any feedback on the current engine torque and dynamics compensation.

For application of the spark ignition (SI) engine model for control purposes, the SI combustion model should strike a balance between model fidelity and computational cost [27]. There have been various types of combustion modeling, which can be grouped into non-predictive models, such as the Wiebe function, and predictive models [28]. Non-predictive models impose a burn rate as a function of crank angle, which means that the burn rate will not be affected by factors such as residual fraction or injection timing. A predictive combustion model would be a good choice for control application because the burn rate will respond appropriately to changes in the variable of interest. One predictive combustion model, the SI turbulent model, predicts the burn rate for homogeneous charges and plays a crucial role in the control application. Even in the high nonlinear conditions where the engine equipped the EGR, water injection, and hydrogen enriched control, the SI turbulent model showed a great fidelity with the computational efficiency [29,30]. The SI turbulent model comprises two subparts of laminar flame speed calculation and entrainment/burn-up. The laminar flame speed is calculated as a function of the equivalence ratio, pressure, temperature, fuel type and dilution effect [31]. The rate of entrained and unburned mixture of fuel and air passing into the flame front through the flame area is proportional to the sum of the turbulent and laminar flame speeds. The burn rate is proportional to the amount of unburned mixture behind the flame front divided by a time constant. This model requires calibration of the four dominant factors of dilution, turbulent flame speed, Taylor microscale length, and flame kernel growth [32,33,34].

In this study, we propose an in-cylinder pressure based 48V mild-hybrid starter-generator (MHSG) control strategy to reduce engine torque deviation under CDA operations. We use commercial software and combine the SI turbulent model for engine modeling with the 48V MHSG model with actual part specifications. In contrast to conventional CDA valve-train systems, the target engine implements CDA with a novel structure of separated intake and exhaust manifolds [35]. To cope with the complexity of the specialized CDA and 48V electrification, the target engine model was first derived using the SI turbulent model based on actual computer-aided design (CAD) files and engine experimental data. The four parameters of the SI turbulent model were calibrated with the in-cylinder pressures obtained by the actual engine experiment. The engine experiment was conducted in 50 operating points, and 100 cycles of in-cylinder pressure data were obtained for each operating condition. The SI turbulent engine model was designed with a strong correlation of representative air states, cylinder pressure, and engine torque. With this engine model, the proposed algorithm calculates instantaneous torque profiles of individual cylinders using in-cylinder pressure traces. Based on the dynamic compensation, the torque setpoint of the 48V MHSG is derived to modulate the slow characteristics of the 48V MHSG from the inherent time delay and response. This is a feedforward control to allow precise control performance with a fast response on a cycle-by-cycle basis for practical torque assist with the 48V MHSG. Moreover, the proposed control structure, where the engine torque is calculated in real-time based on the in-cylinder pressure, effectively reduces the effort needed to calibrate the conventional map-based open-loop controller under various types of engine operations. Furthermore, the proposed algorithm effectively reduces engine torque deviation under the main driving range of the target CDA operations.

## 2. Experimental Setup

### 2.1. Target Engine Description

**Figure 1:**
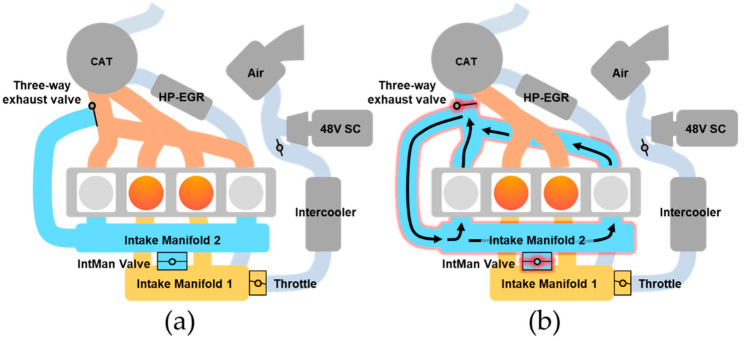
Operations of the target engine: (**a**) Four-cylinder operation; (**b**) Two-cylinder operation.

The target engine was a 1.6 L gasoline direct injection (GDI) engine, designed with a specialized architecture of separated intake and exhaust manifolds to accommodate the CDA operation [35]. The detailed working principle of this particular CDA is illustrated in Figure 1. Intake manifold 1 is connected to cylinders 2 and 3. In the same manner, intake manifold 2 is linked to cylinders 1 and 4 to form two different closed airflow loops. These two independent loops allow the engine to operate with four or two cylinders through control of the intake manifold valve and the three-way exhaust valve.

As described in Figure 1a, four-cylinder operation is realized by fully opening the intake manifold valve between the separated intake manifolds and closing the three-way exhaust valve. Except for small cylinder-to-cylinder variations from the air flow through an additional throttle, the engine operates as a normal four-cylinder engine. On the other hand, under two-cylinder operation, as shown in Figure 1b, the intake manifold valve is closed to cut off airflow into intake manifold 2 and the three-way exhaust valve is opened to isolate the internal loop of cylinders 1 and 4. This enables deactivation of cylinders 1 and 4, along with the prevention of fuel injection and ignition. Since the closed loop of the deactivated cylinders does not involve a pressure difference between intake and exhaust strokes, the pumping loss can be eliminated simultaneously.

The target engine is equipped with external high-pressure exhaust gas recirculation (HP-EGR), variable valve timing (VVT), an intercooler, and a 48V electric supercharger, as shown in Table 1. The engine is also equipped with a 48V MHSG, which is connected by a pulley to assist in generating engine power; detailed specifications are provided in Table 2.

### 2.2. Experimental Conditions

**Figure 2:**
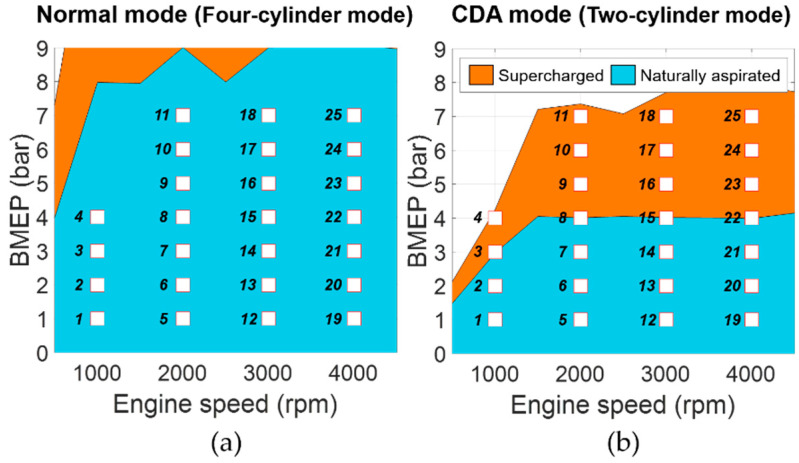
Engine operating conditions: (**a**) Normal mode; (**b**) cylinder deactivation (CDA) mode.

The target engine was modeled with actual experimental engine data from 50 steady states. Figure 2 shows the steady states, represented as value of brake mean effective pressure (BMEP) and engine speed. The normal mode of four-cylinder operation includes a total of 25 naturally aspirated regions, whereas the CDA mode contains 15 naturally aspirated and 10 supercharging areas to track the target BMEP values in only two-cylinder operation. To derive a precise combustion model, 100 cycles of in-cylinder pressure data were obtained for each operating condition. The measured pressure signals were sampled every 1 crank angle degree. Depending on the two-cylinder or four-cylinder operation, each spark timing map has been calibrated to produce maximum brake torque to reflect the trapped mass influence caused by the different activated cylinder configuration. The HP-EGR rate, VVT, spark timing, and intercooler temperature set-points were fixed at specific target values for each engine operating point.

## 3. Target Engine Model and 48V MHSG Model

### 3.1. Model Overview

**Figure 3:**
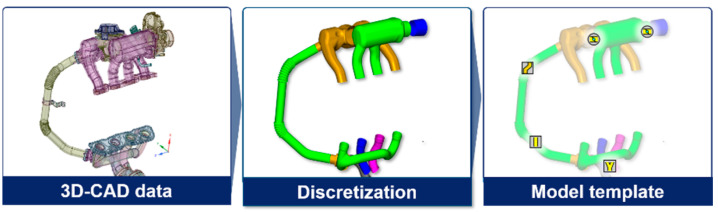
Process of importing 3D computer-aided design (CAD) files with discretization.

**Figure 4:**
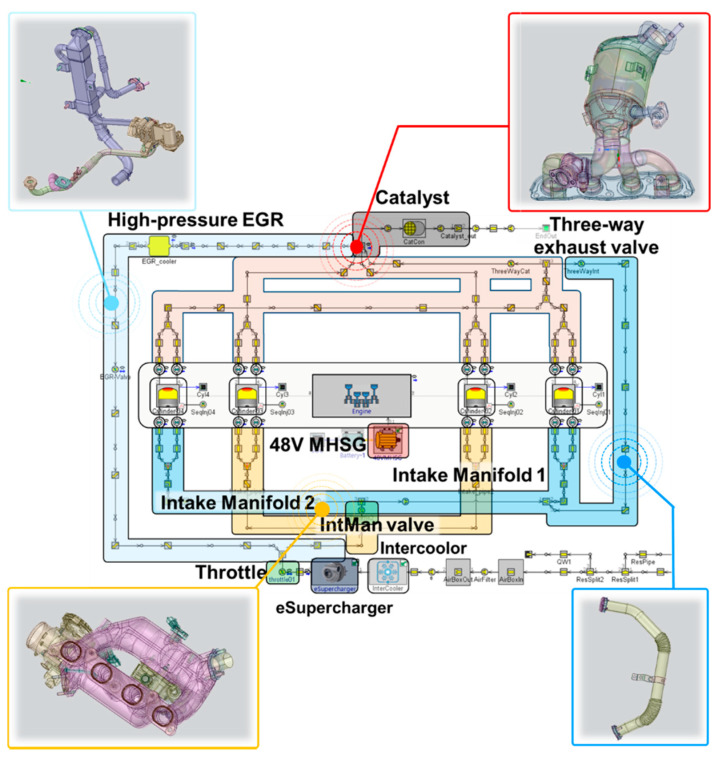
Schematics of the target engine model.

To deal with the enormous complexity of cylinder deactivation combined with a 48V mild hybrid system, the target engine model was designed using GT-POWER simulation. In particular, the simulation model was derived based on 3D CAD files to precisely reflect the geometric information of the specialized engine structure. Figure 3 represents the process of importing the actual 3D CAD files into the simulation environment. The discretization process divides a large volume assembly into smaller connected subcomponents. After the ensuing procedure of simplification to eliminate unnecessary obstacles with respect to the model accuracy, the simulation model analyzes the intake and exhaust flow rates based on 1D fluid dynamics. The forward and backward discharge coefficients were addressed based on the unit experimental test for components of valve types, such as throttle, intake manifold valve, and three-way exhaust valve. The 48V electric supercharger and MHSG were also implemented with the specification values, as shown in the overall model schematics in Figure 4.

### 3.2. SI Turbulent Engine Model

The in-cylinder pressure is essential information indicating the combustion status of individual cylinders in CDA operations; therefore, the engine model was precisely designed using the SI turbulent flame model of GT-POWER to reflect various in-cylinder conditions such as cylinder geometry, spark location, and mixture characteristics. The SI turbulent flame model calculates the entrained mass of mixture ($M_{e}$) from a rate proportional to the sum of the turbulent flame speed ($S_{T}$) and the laminar flame speed ($S_{L}$). The derivative of burned mass ($M_{b}$) is formulated by dividing the difference of $M_{e}$ and $M_{b}$ by the time constant of the burn rate ($\tau_{b}$), as follows [28,36]:

(1) $$\frac{dM_{e}}{dt}=\;\rho_{u}A_{e}\left(S_{T}+S_{L}\right),$$

(2) $$\frac{dM_{b}}{dt}=\;\frac{M_{e}-M_{b}}{\tau_{b}},$$

where $\rho_{u}$ is the unburned mixture density, and $A_{e}$ is the surface area at the flame front location.

The four dominant phenomena of dilution, turbulent flame speed, Taylor microscale length, and flame kernel growth determine the flame model [32,33,34]. The dilution effect of residuals and external EGR is sophisticatedly calibrated with the dilution multiplier ($C_{DE}$). The $C_{DE}$ scales the dilution effect term ($E_{D}$) with the burned gas fraction in the unburned zone ($f_{b}$). $E_{D}$ is used as a proportional factor to determine $S_{L}$ based on the maximum laminar speed ($B_{m}$), laminar speed roll-off value ($B_{\phi }$), equivalence ratio (ϕ), and equivalence ratio at maximum speed ($\phi_{m}$), with the following formula:

(3) $$E_{D}=1-0.75\cdot C_{DE}\cdot \left(1-{\left(1-0.75\cdot C_{DE}\cdot f_{b}\right)}^{7}\right),$$

(4) $$S_{L}=\left(B_{m}+B_{\phi }{\left(\phi -\phi_{m}\right)}^{2}\right)\cdot {\left(\frac{T_{u}}{T_{ref}}\right)}^{\alpha }\cdot {\left(\frac{p}{p_{ref}}\right)}^{\beta }\cdot E_{D},$$

where p is the pressure, $T_{u}$ is the unburned gas temperature, α and β are temperature exponent and pressure exponent values, and $T_{ref}$ and $p_{ref}$ are constant values to make p and $T_{u}$ dimensionless.

The flame kernel growth multiplier ($C_{FKG}$) and the turbulent flame speed multiplier ($C_{TFS}$) represent magnitude factors of the turbulent flame speed. $C_{FKG}$ has an especially large influence on the initial combustion velocity with the smaller flame radius ($R_{f}$), as follows:

(5) $$S_{T}=C_{TFS}u^{\prime }\left(1-\frac{1}{1+C_{FKG}{\left(\frac{R_{f}}{L_{i}}\right)}^{2}}\right),$$

where $u^{\prime }$ is the turbulent intensity, and $L_{i}$ is the integral length scale.

The Taylor length scale multiplier ($C_{TLS}$) is a parameter that modulates the propagation speed of the burn rate with respect to the Taylor microscale length (λ). The λ is calculated based on $L_{i}$ and the turbulent Reynolds number ($Re_{t}$). The $C_{TLS}$ factor is multiplied to calibrate the degree of Taylor length scale for the flame propagation:

(6) $$\lambda =\frac{C_{TLS}L_{i}}{\sqrt{Re_{t}}},$$

(7) $$\tau_{b}=\frac{\lambda }{S_{L}},$$

To calibrate the combustion model, in-cylinder pressure measurements of the engine experimental data were utilized. The goal of combustion model calibration is to determine the best set of four scale multipliers $C_{DE}$, $C_{FKG}$, $C_{TFS}$, and $C_{TLS}$. The optimal set was derived based on a grid search method using in-cylinder pressure measurements for a total of 50 engine operating conditions.

### 3.3. 48V MHSG Model

**Figure 5:**
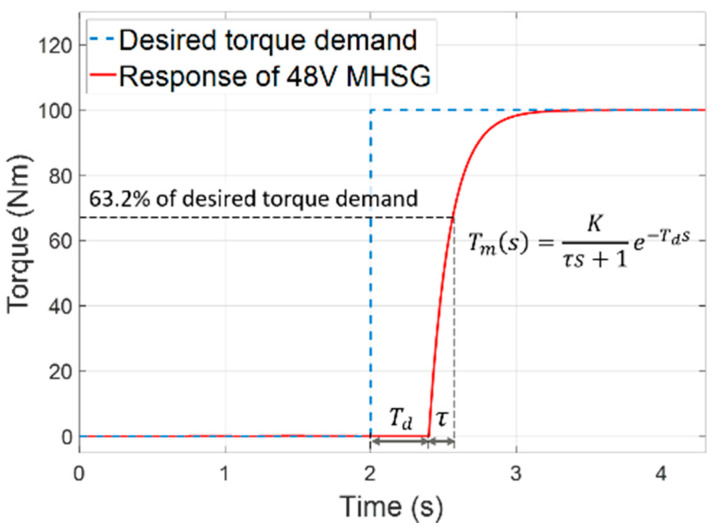
Modeling of the 48V mild-hybrid starter-generator (MHSG) with first-order dynamic response.

From a control point of view, it is important to accurately model the dynamic characteristics to adequately perform the torque assist function of the 48V MHSG. The inherent electromagnetic and mechanical features cause specific response times. Moreover, time delay also occurs depending on certain periods of the internal control modules and communication events. As shown in Figure 5, these dynamic characteristics were implemented using a first-order system with a time delay:

(8) $$T_{m}\left(s\right)=\;\frac{K}{\tau s+1}e^{-T_{d}s},$$

where the parameter K is the DC gain. The time constant (τ) and time delay ($T_{d}$) were generated with component specification values using a look-up table (LUT) for the current engine operating conditions. The 48V MHSG model was connected to the engine flywheel with a certain pulley ratio, rated power, and torque specifications as shown in Table 2.

### 3.4. Modeling Results

**Figure 6:**
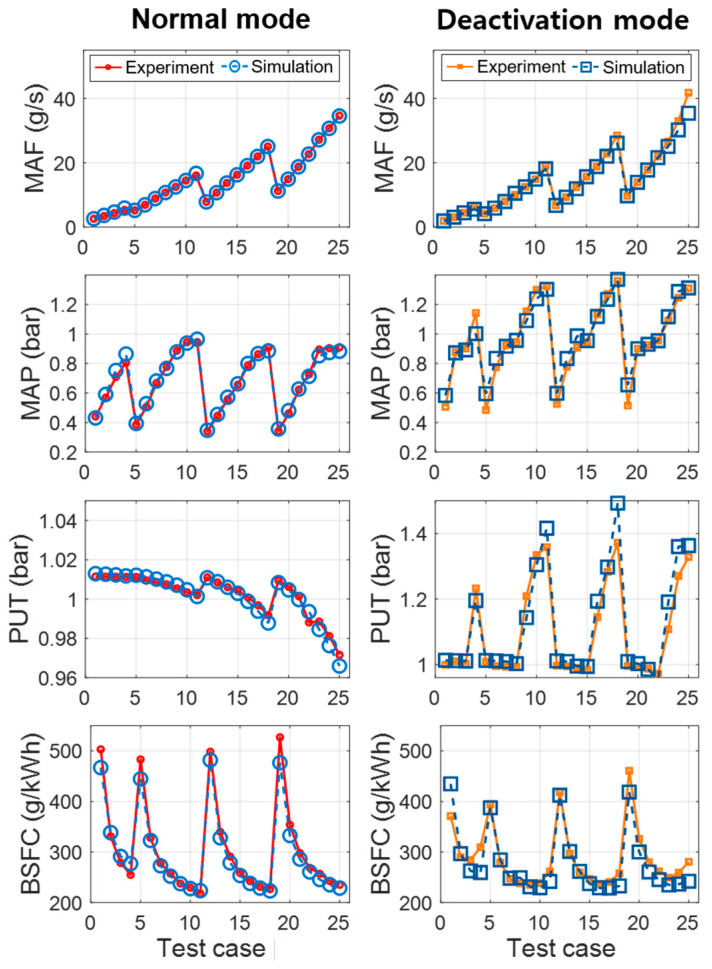
Modeling results of the air states and brake specific fuel consumption (BSFC).

Figure 6 provides a comparison of the simulation results and engine experimental measurements under four-cylinder and two-cylinder engine operations. The four figures show the mass air flow (MAF), manifold absolute pressure (MAP), pressure at upstream of throttle (PUT), and brake specific fuel consumption (BSFC). The performance indices of the coefficient of determination ($R^{2}$) and root mean squared error (RMSE) are also summarized in Table 3. The representative air states of MAF, MAP, and PUT showed a strong correlation, with R^2^ values over 0.97. BSFC, a performance indicator of fuel consumption, also presented an accurate trend, with an R^2^ value over 0.94 and RMSE value less than 2.4 g/kWh.

**Figure 7:**
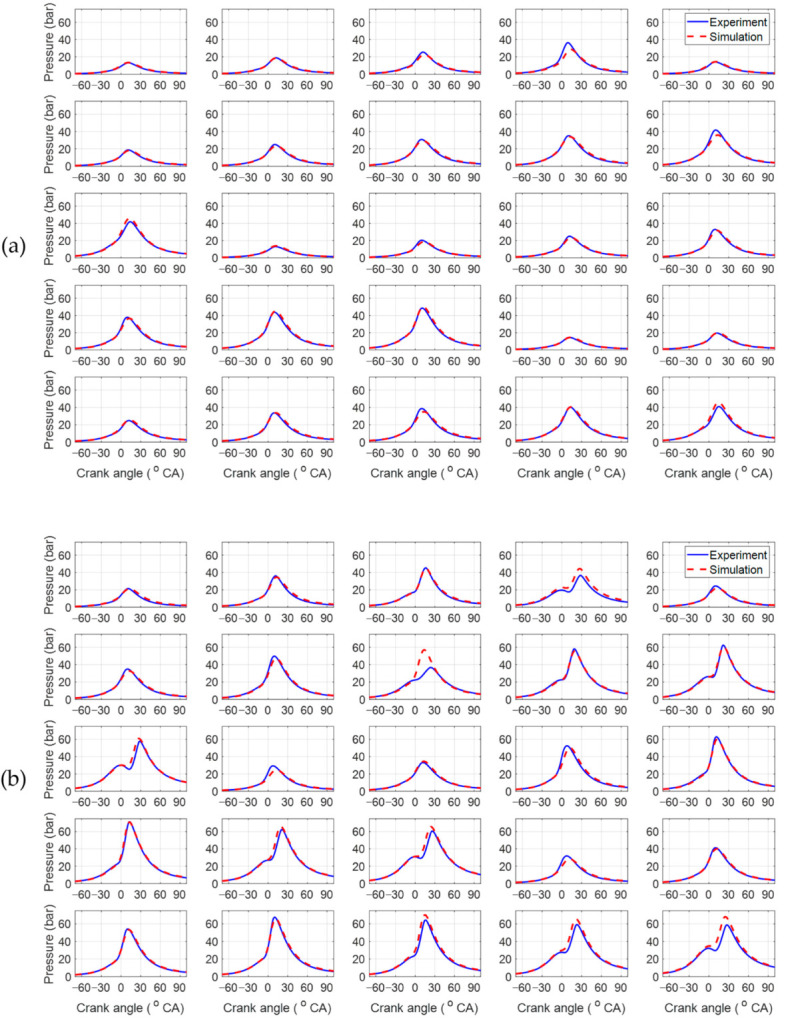
Comparison of the in-cylinder pressure traces: (**a**) four-cylinder operation; (**b**) two-cylinder operation.

**Figure 8:**
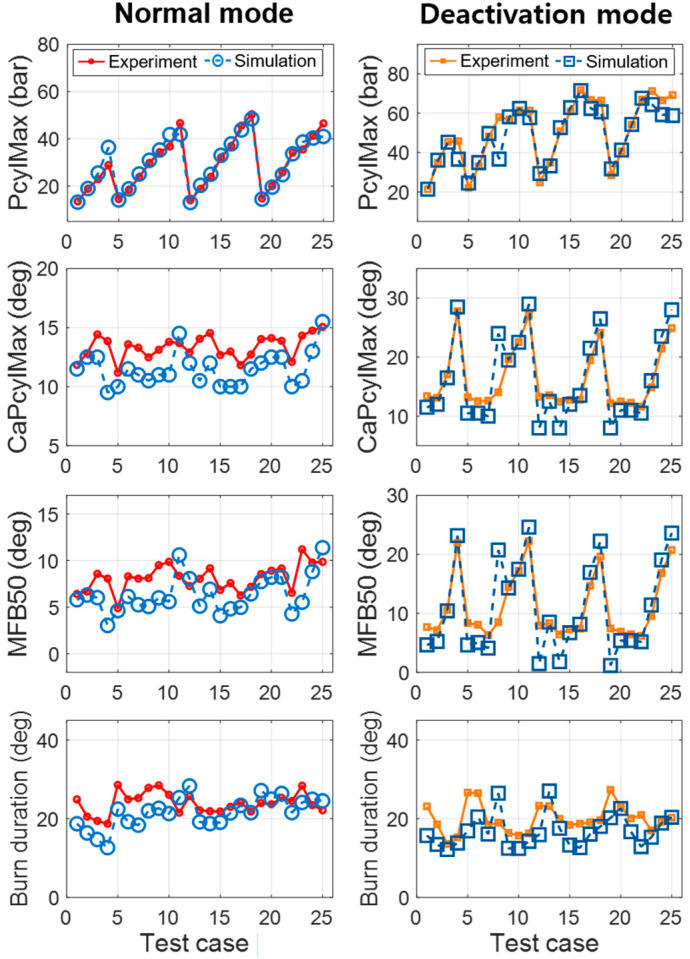
Modeling results of the combustion characteristics.

Figure 7 show modeling results of in-cylinder pressure traces in normal and CDA modes, respectively. These figures show fitted results of in-cylinder pressure from the combustion model over a wide range of engine operations. For quantitative comparison of the combustion models, the combustion characteristics were calculated from the cylinder pressure, as shown in Figure 8 and Table 4. The maximum value of pressure trace (PcylMax) and its crank angle location (CaPcylMax) had values of less than 0.6 bar and 0.3 degree, respectively, showing notable RMSE accuracy. The crank angle location of the mass fraction burned 50% (MFB50) and the burn duration difference between MFB90 and MFB10 (MFB90-MFB10) were also precise, with RMSE values of less than 0.5 degree.

**Figure 9:**
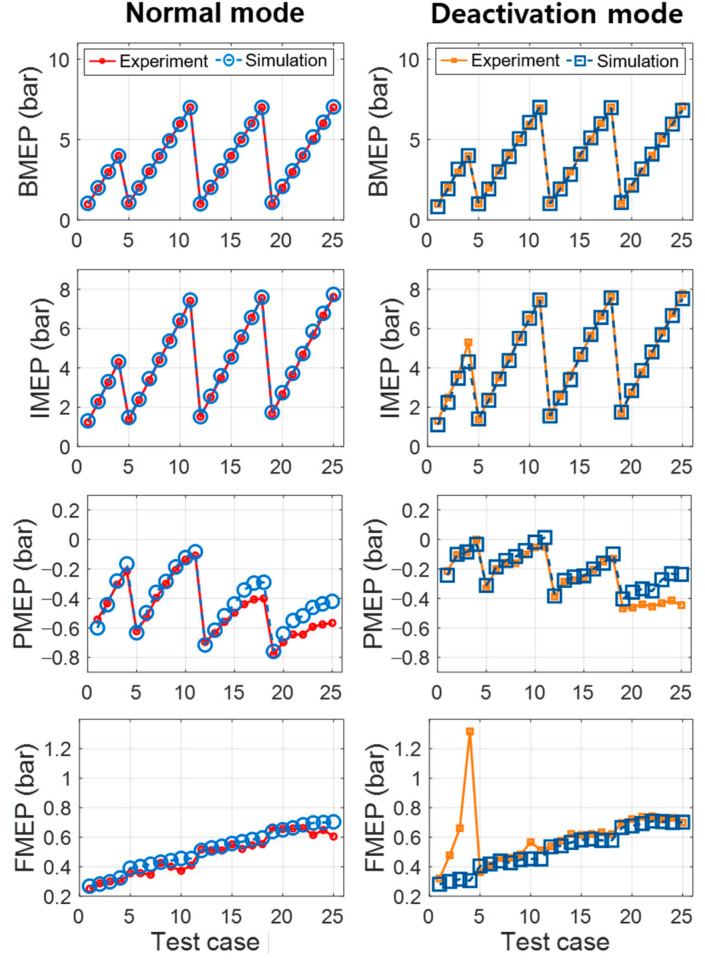
Modeling results of the mean effective pressure values.

The mean effective pressure values of BMEP, indicated mean effective pressure (IMEP), pumping mean effective pressure (PMEP), and friction mean effective pressure (FMEP) were also analyzed, as shown in Figure 9. BMEP and IMEP, which denote the engine brake torque and indicated combustion torque, showed precise modeling results with R^2^ values over 0.99 and RMSE values under 0.04 bar, as detailed in Table 5. Both PMEP and FMEP were also calculated with RMSE values below 0.03 bar. However, FMEP at some operating points showed a discrepancy compared to the experiment. It is caused by the limitation of the linear regression model to estimate the FMEP. The regression model was based on a least-square method, called the Chen-Flynn engine friction model [28]. Overall, these results indicate that the engine model accurately simulates the various engine conditions under normal and CDA operations.

## 4. In-Cylinder Pressure Based 48V MHSG Control Strategy

### 4.1. Controller Overview

**Figure 10:**
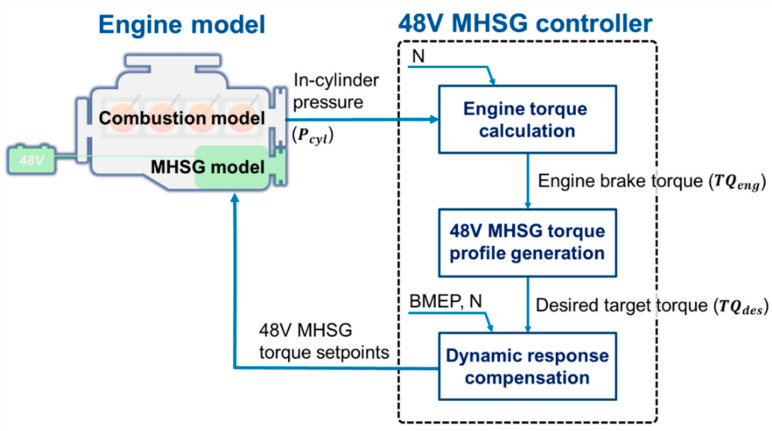
Overall process of the proposed 48V MHSG control strategy.

The design objective of the proposed algorithm is to precisely control the 48V MHSG to reduce engine torque deviation under CDA operations. The control algorithm was designed based on the engine model to efficiently handle the high complexity of the target system. Figure 10 shows the overall architecture of the proposed in-cylinder pressure based 48V MHSG controller. The controller was composed of (1) engine torque calculation, (2) 48V MHSG torque profile generation, and (3) dynamic response compensation. The control input of the controller is the in-cylinder pressure in the previous engine cycle, and it can calculate the real-time engine torque in each cylinder. In addition, the target torque profile of the 48V MHSG is generated using the torque deviations derived from the difference between the activated and deactivated cylinders. In more detail, the desired 48V MHSG torque is calculated every 0.5 degree of the crank angle within one engine cycle. The desired target torque profile can minimize the engine torque deviation in an ideal situation. However, the control strategy requires a rapid response to control the 48V MHSG torque in the crank angle domain. Otherwise, the dynamic response of 48V MHSG could distort the torque profile. Therefore, the last step of dynamic compensation finally produces the torque set-point to modulate the time delay and transient response of the 48V MHSG. To alleviate the time delay, the target profile is shifted to match the control instance to the next complete engine cycle. The transient response, which is approximated by the first-order time constant, compensated by a lead compensator which places a dominant pole to the point where has fast response characteristics.

### 4.2. Engine Torque Calculation

**Figure 11:**
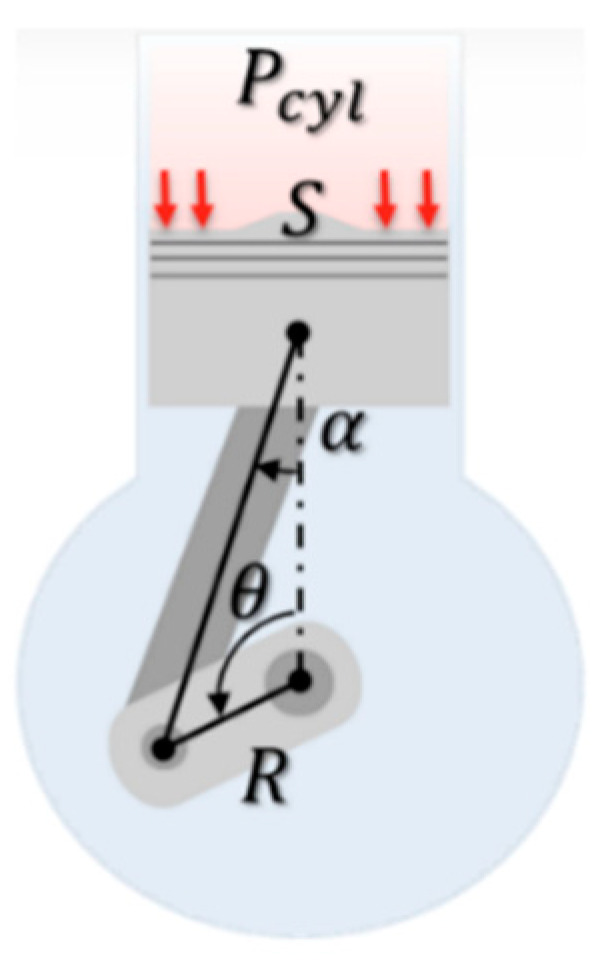
In-cylinder pressure and cylinder geometry.

The in-cylinder pressure is important information that allows instantaneous torque calculation for the individual cylinders. As described in Figure 11, the indicated torque of the individual cylinders is calculated using the in-cylinder pressure ($P_{cyl}$) and the geometry. The total indicated torque applied to the crankshaft $TQ_{ind}\left(\theta \right)$ is formulated for each crank angle (θ) as follows:

(9) $$TQ_{ind}\left(\theta \right)=\underset{n_{c}}{\sum }P_{cyl}\left(\theta \right)\cdot S\cdot R\cdot sin\left(\theta \right)-P_{cyl}\left(\theta \right)\cdot S\cdot R\cdot cos\left(\theta \right)\cdot tan\left(\alpha \right),$$

where $n_{c}$ is the number of cylinders, and S, R, and α are the cross-sectional area of the piston, length of the crank, and connecting rod angle, respectively.

The inertia torque $TQ_{iner}\left(\theta \right)$ is the part of the engine torque generated from the crankshaft moment of inertia. The formulation is derived as a negative sign with multiplication of the effective inertia moment ($I_{eff}$) and derivative of the instantaneous engine speed ($\dot{N}$):

(10) $$TQ_{iner}\left(\theta \right)=-I_{eff}\cdot \frac{2\pi }{60}\cdot \dot{N}\left(\theta \right),$$

The friction torque $TQ_{fric}$ is approximated as a mean value of one engine cycle. The FMEP is calculated based on the Chen-Flynn engine friction model [28] with a polynomial equation of the PcylMax and a cycle mean value of the engine speed ($\bar{N}$), as follows:

(11) $$\mathrm{FMEP}=C_{0}+C_{1}\cdot \mathrm{PcylMax}+C_{2}\cdot \bar{N}+C_{3}\cdot {\bar{N}}^{2},$$

(12) $$TQ_{fric}=-\frac{n_{c}V_{d}}{2\pi n_{r}}\cdot \mathrm{FMEP},$$

where $n_{r}$ is the number of crankshaft rotations for a complete engine cycle, and $V_{d}$ is the engine displacement volume. The coefficients $C_{0}$, $C_{1}$, $C_{2}$, and $C_{3}$ are derived using the least-square method.

**Figure 12:**
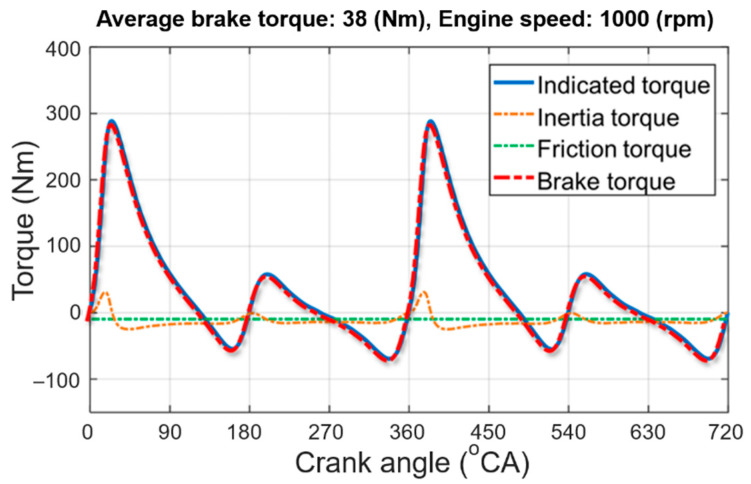
Calculated engine torques in crank angle domain.

As shown in Figure 12, the engine brake torque $TQ_{eng}\left(\theta \right)$ is calculated as the sum of $TQ_{ind}\left(\theta \right)$, $TQ_{iner}\left(\theta \right)$, and $TQ_{fric}$ with the following equation:

(13) $$TQ_{eng}\left(\theta \right)=TQ_{ind}\left(\theta \right)+TQ_{iner}\left(\theta \right)+TQ_{fric},$$

### 4.3. 48V MHSG Torque Profile Generation

**Figure 13:**
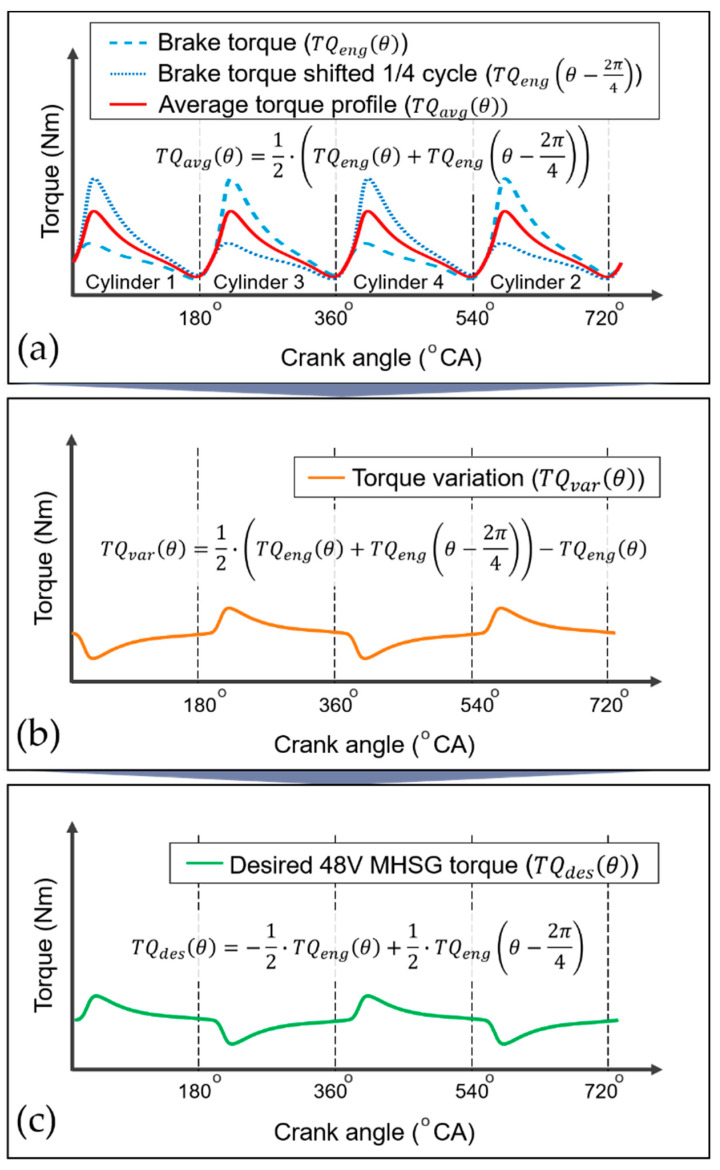
Calculation of the desired 48V MHSG torque profile in crank angle domain: (**a**) Average torque; (**b**) Torque variation; (**c**) Desired 48V MHSG torque.

Based on the calculated engine brake torque, the desired torque profile of the 48V MHSG is generated to eliminate engine torque deviation at each crank angle. Since the sequence of activation and deactivation is repeated every 1/4 cycle in a four-cylinder engine, the deviation can be derived from the brake torque trace shifted by 1/4 cycle, as illustrated in Figure 13. Therefore, the desired profile for the torque assist with 48V MHSG is produced as follows:

(14) $$TQ_{des}\left(\theta \right)=\frac{1}{2}\cdot \left(TQ_{eng}\left(\theta \right)+TQ_{eng}\left(\theta -\frac{2\pi }{4}\right)\right)-TQ_{eng}\left(\theta \right)\;=-\frac{1}{2}\cdot TQ_{eng}\left(\theta \right)+\frac{1}{2}\cdot TQ_{eng}\left(\theta -\frac{2\pi }{4}\right)$$

### 4.4. Dynamic Compensation of 48V MHSG

**Figure 14:**
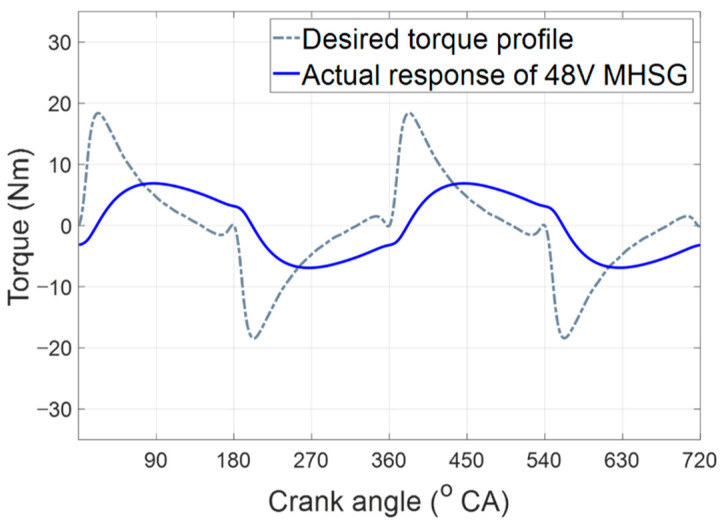
Actual response of the 48V MHSG without dynamic compensation.

Accurate torque assist requires a rapid response of 48V MHSG on a cycle-by-cycle basis; however, the inherently slow characteristics and time delay of the 48V MHSG deteriorate the control accuracy for tracking the target torque waveform. Figure 14 shows the distorted torque waveform without any compensation of dynamic response. The grey dashed line is desired target torque profile, and the blue line is actual torque trajectory which cannot tracking the target torque profile.

**Figure 15:**
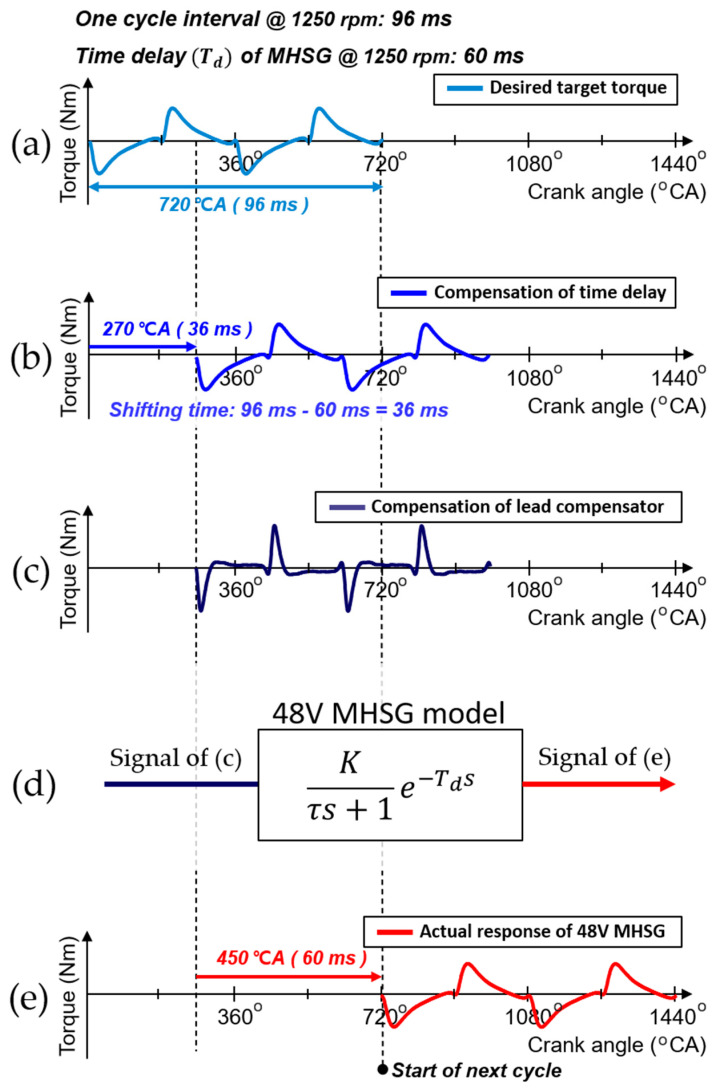
Procedures of the dynamic compensation for time delay and time constant: (**a**) Desired torque; (**b**) Compensation of time delay; (**c**) Compensation of lead compensator; (**d**) Application of dynamic response in 48V MHSG; (**e**) Actual response of 48V MHSG.

To resolve this problem, the setpoint applied to the 48V MHSG is finally modulated through dynamic compensation of the desired torque profile. The controller already has two dynamic characteristic parameters as a LUT for the current engine operating conditions: the time delay ($T_{d}$) and time constant (τ) explained in Equation (8). Figure 15 shows the sequential procedures for the dynamic compensation with the parameters. The signal in Figure 15a represents a desired torque profile of the 48V MHSG. In ideal conditions, the desired torque profile can minimize the engine torque deviation from the CDA. Each signal in Figure 15b,c shows the control instance after the compensation of the $T_{d}$ and τ, respectively. After both compensations, the control instance is applied to the 48V MHSG which has a dynamic response as shown in Figure 15d. Through the compensation, the actual torque profile in Figure 15e which is generated from the 48V MHSG is almost identical to the desired torque profile.

In the case of the $T_{d}$ compensation, the delay effect is mitigated by matching the control instance to the next complete engine cycle. The target profile is further shifted by the difference between the 48V MHSG time delay and the current cycle interval, calculated from the instantaneous engine speed. For the compensation of the τ, the phase-lead compensator is designed to modulate the slow dynamic characteristics. The phase-lead compensator contributes one zero and one pole to the system. The additional zero eliminates the slow response of MHSG, which is approximated as a first-order time constant τ, through the pole-zero cancellation. And the additional pole improves the response characteristic of the system as a new dominant pole. The formula of the phase-lead compensator is as follows:

(15) $$T_{c}\left(s\right)=K_{c}\frac{\tau \cdot s+1}{\omega \cdot \tau \cdot s+1},$$

where the $K_{c}$ is control gain to compensate a steady-state error and parameter ω is an attenuation factor of the lead compensator which has a value between 0 to 1. The compensator contributes one zero and one pole to the system at −1/τ and −1/ωτ, respectively. After the initial value search based on the pole-zero cancelation, the $K_{c}$ and ω values fine-tuned to offset the time constant of the 48V MHSG specifications. These values are also generated as a LUT according to the current engine operating condition.

### 4.5. 48V MHSG Control Results

**Figure 16:**
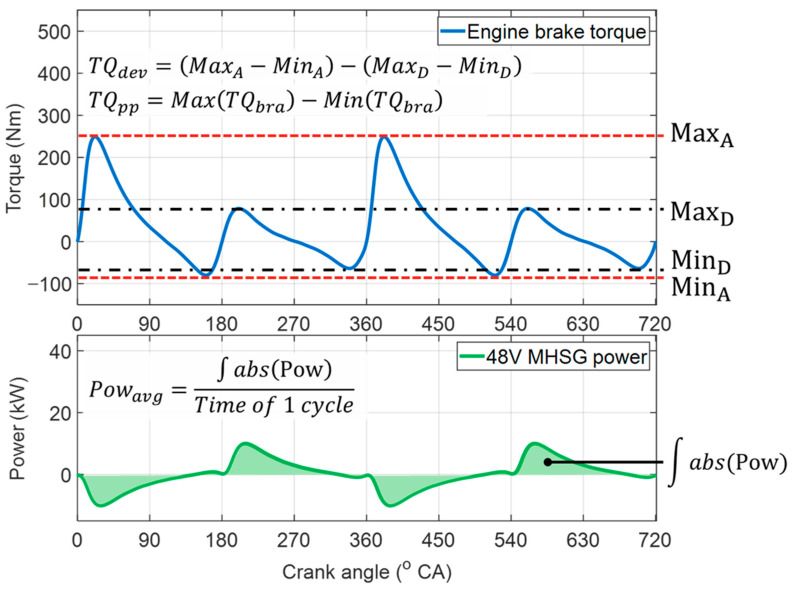
Performance index to evaluate the proposed control strategy.

**Figure 17:**
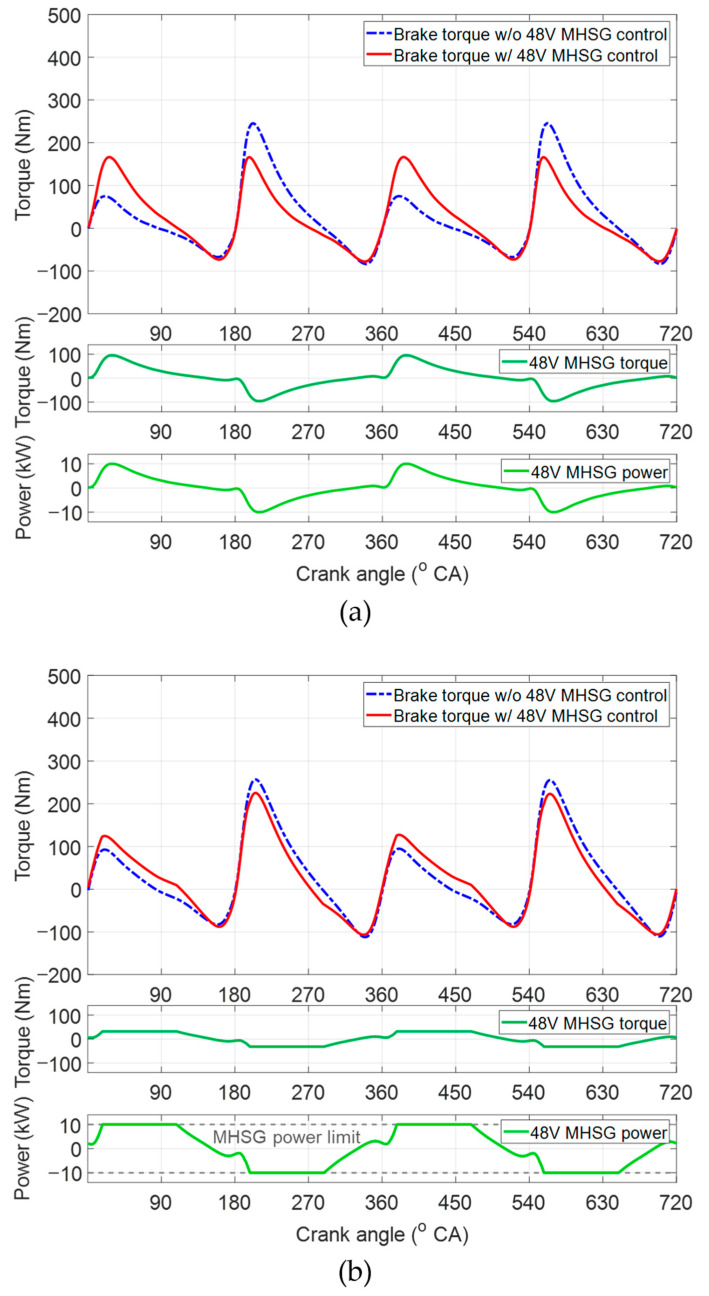
48V MHSG control results: (**a**) engine speed of 1000 rpm and brake mean effective pressure (BMEP) of 2 bar; (**b**) engine speed of 3000 rpm and BMEP of 2 bar.

As shown in Figure 16, the performance of the proposed control algorithm was evaluated according to the reduction rates of the torque deviations, peak-to-peak values of the engine torque traces, and average power of the 48V MHSG without any regeneration. Figure 17a shows the control results for 48V MHSG at constant engine speed of 1000 rpm, BMEP of 2 bar. Compared to the results without 48V MHSG torque assistance, the proposed algorithm reduced the engine torque deviation by 98% and the peak-to-peak value by 26%. In this operating area, the average power of 48V MHSG was 3.54 kW. These results demonstrate that the proposed control algorithm effectively reduces torque fluctuation between the activated and deactivated cylinders under CDA operations. Moreover, they indicate that the specifications of 48V MHSG are sufficient to mitigate torque variations in this engine operating condition, with working range of the 48V MHSG torque and power within rated values of 100 Nm and 10 kW. On the other hand, the results presented in Figure 17b show relatively small reduction rates of 38% and 10% in the deviation and peak-to-peak indices, despite the MHSG consumes a larger average power of 6.9 kW. This is because torque variation was not completely eliminated in this engine operating area at an engine speed of 3000 rpm and BMEP of 2 bar, due to the limited power of the 48V MHSG.

**Figure 18:**
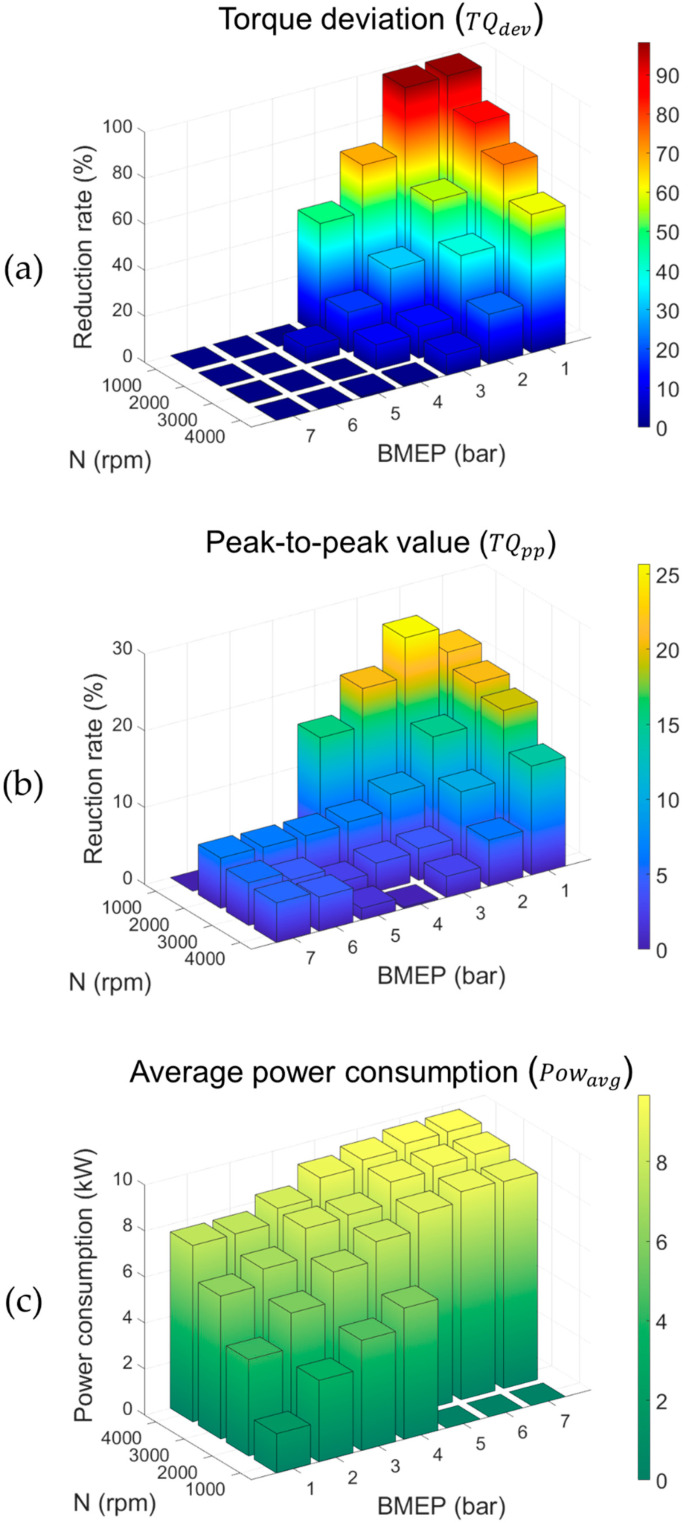
Reduction rate with respect to engine operating conditions: (**a**) the torque deviations; (**b**) the peak-to-peak values; (**c**) the average power consumptions.

Figure 18 summarize the control results for each engine operating condition. Torque deviations were calculated with a maximum decrease rate of 98% at an engine speed of 1000 rpm and BMEP of 2 bar, and a minimum reduction rate of 0.1% in the engine operation at an engine speed of 4000 rpm and BMEP of 7 bar. For the reduction rate in peak-to-peak values, a maximum of 26% and minimum of 0.2% were derived under operation with an engine speed of 1000 rpm, BMEP of 2 bar, area at engine speed of 4000 rpm, and BMEP of 4 bar. The average power of the 48V MHSG was proportional to engine speed and BMEP in the range of 1.71 kW to 9.67 kW, and it converged to 48V MHSG’s maximum power of 10 kW. These analysis results indicate that the proposed algorithm practically decreases the torque deviations of the CDA engine with 48V MHSG specifications, mainly under low engine speed and load conditions. In other words, the proposed controller provides effective performance under the primary target driving area of CDA operations.

## 5. Conclusions

In this paper, an in-cylinder pressure based 48V MHSG control strategy was proposed for minimizing engine torque deviation in the CDA engine. To deal with the significant complexity of the integrated system of CDA and mild-hybrid electrification, the proposed control algorithm was designed in a simulation environment.

The SI turbulent engine model was derived based on 3D CAD data to implement the geometric information of the particular structure with a separated intake and exhaust manifolds. The model included a combustion model that was finely calibrated using in-cylinder pressure measurements, and a 48V MHSG model with dynamic characteristics based on detailed specifications. The modeling results of BMEP and IMEP showed a precise accuracy, with RMSE values under 0.04 bar. PcylMax and CaPcylMax presented RMSE values of less than 0.6 bar and 0.3 degree, respectively.

The performance of the proposed 48V MHSG control algorithm was analyzed with respect to the engine operating range. The reduction rate of the torque deviations and peak-to-peak values were calculated and found to have maximum values of 98% and 26%, respectively. At the maximum reduction rate the average power of 48V MHSG was 3.54 kW without any regeneration. These results demonstrate that the proposed strategy effectively reduced engine torque deviations through accurate control of the 48V MHSG, under target CDA operations of low engine speed and load conditions.

As a follow-up research, the proposed 48V MHSG control algorithm which was evaluated through a simulation will be implemented into an actual vehicle for the investigation of NVH issues during the mode transition such as four-cylinder to two-cylinder mode transition, and vice versa. In addition, battery management considering regenerative braking will be considered to analyze fuel efficiency while running specific driving cycles such as NEDC and WLTP.

## Figures and Tables

**Table 1:** Specifications of target engine.

Description	Specifications
Engine type	Gasoline direct injection (GDI), Inline, Double overhead camshaft (DOHC)
Number of cylinders	4
Bore × stroke (mm)	72 × 97
Connecting rod (mm)	152.2
Displacement volume (L)	1.580
Compression ratio	12:1
Applied technologies	Cylinder deactivation (CDA) with separated manifolds architecture
	External high-pressure EGR (HP-EGR)
	Intake/exhaust variable valve timing (VVT)
	Intercooler
	48V electric supercharger
	48V mild-hybrid starter-generator (MHSG)

**Table 2:** Specifications of 48V MHSG.

Description	Specifications
Pulley ratio	2.67
Maximum and minimum power	±10 kW
Maximum and minimum torque	±100 Nm

**Table 3:** Performance index of the modeling results for air states and BSFC: the coefficient of determination ($R^{2}$) and root mean squared error (RMSE).

	Normal Mode (Four-Cylinder Operation)		Deactivation Mode (Two-Cylinder Operation)	
	$R^{2}$	RMSE	$R^{2}$	RMSE
MAF (g/s)	0.999	0.019	0.995	0.154
MAP (bar)	0.994	0.002	0.983	0.006
PUT (bar)	0.986	0.002	0.973	0.004
BSFC (g/kWh)	0.993	1.748	0.941	2.363

**Table 4:** Performance index of the modeling results for combustion characteristics.

	Normal Mode (Four-Cylinder Operation)		Deactivation Mode (Two-Cylinder Operation)	
	$R^{2}$	RMSE	$R^{2}$	RMSE
PcylMax (bar)	0.972	0.261	0.939	0.590
CaPcylMax (deg)	0.560	0.221	0.924	0.299
MFB50 (deg)	0.424	0.253	0.901	0.365
Burn duration (deg)	0.500	0.410	0.529	0.484

**Table 5:** Performance index of the modeling results for mean effective pressure values.

	Normal Mode (Four-Cylinder Operation)		Deactivation Mode (Two-Cylinder Operation)	
	$R^{2}$	RMSE	$R^{2}$	RMSE
BMEP (bar)	0.999	0.005	0.998	0.011
IMEP (bar)	0.999	0.014	0.994	0.037
PMEP (bar)	0.949	0.008	0.861	0.013
FMEP (bar)	0.973	0.004	0.260	0.022

## Data Availability

Not applicable.
